# Inequalities in disease burden and care quality of neglected tropical diseases and malaria, 1990–2021: Findings from the 2021 Global Burden of Disease Study

**DOI:** 10.1371/journal.pone.0329475

**Published:** 2025-08-07

**Authors:** Ran He, Wenhao Zhu, Shirong Hui, Meijie Yu, Haochang Li, Yihao Li, Peng Huang, Rongbin Yu

**Affiliations:** Department of Epidemiology, National Vaccine Innovation Platform, Centre for Global Health, School of Public Health, Key Laboratory of Public Health Safety and Emergency Prevention and Control Technology of Higher Education Institutions in Jiangsu Province, Nanjing Medical University, Nanjing, China; Utkal University, INDIA

## Abstract

**Background:**

Neglected Tropical Diseases (NTDs) and malaria represent significant public health threats, particularly in regions with low Socio-Demographic Index (SDI) levels. Despite global efforts to enhance prevention and control, disparities still persist. We aimed to assess the quality of care for NTDs and malaria through the quality of care index (QCI) to analyse differences in regions, genders, age groups, and to provide measures to improve the quality of care to reduce the burden of disease.

**Methods:**

Using data from the Global Burden of Disease Study 2021 (GBD 2021), four secondary indicators were calculated using six primary indicators: incidence, mortality, prevalence, the disability-adjusted life years (DALYs), years of life lost, and years lived with disability to calculate the QCI through principal component analysis, and the gender disparity ratio was the ratio of female QCI to male QCI.

**Results:**

Globally, the incidence of NTDs and malaria increased, while age-standardized mortality rates (ASMRs) and DALYs declined from 1990 to 2021. In 2021, the QCI was highest in Bahrain, Qatar, and the United Arab Emirates, with scores of 91.9, 91.5, and 91.5, respectively. In contrast, Uganda, Niger, and Burkina Faso had the lowest QCIs, at 78.5, 77.1, and 75.2, respectively. QCI showed a positive correlation with a country’s level of economic development, with lower quality of care observed in those under 10 and over 95 years, and largely equal quality of care received between genders.

**Conclusions:**

This study revealed differences in the quality of care for NTDs and malaria across regions, genders, and age groups. There is an urgent need to improve care quality, particularly in low SDI areas and among young children. The study provides recommendations for enhancing the quality of care for NTDs and malaria and for developing more effective public health interventions.

## Introduction

Neglected tropical diseases (NTDs) and malaria pose a major threat to public health globally, especially in low- and middle-income countries. NTDs, which include a range of diseases caused by parasites, bacteria, viruses and fungi, have a high disease burden but are often overlooked in the global health agenda [1]. It is estimated that more than 1 billion people are affected by NTDs globally, while the total number of people in need of preventive and curative interventions is 1.6 billion [2]. Malaria, on the other hand, causes millions of morbidities and deaths each year. For example, in 2022, there were 249 million cases of malaria globally, resulting in 608,000 deaths [3].

NTDs have many overlaps with malaria in terms of geographic distribution, endemic areas and affected groups, affecting mainly poor regions in tropical and subtropical areas [4]. There are also overlaps between NTDs and malaria in terms of disease transmission routes, hosts and vectors [5]. Similar challenges are faced in diagnosis, treatment and vaccine development [6]. Therefore, our study of NTDs together with malaria can help to optimise public health interventions in resource-limited settings, increase attention to these diseases, raise their priority on the global health agenda, and allocate limited health resources more efficiently [7]. At the same time, in order to achieve the United Nations Sustainable Development Goals (SDGs) for ‘good health and well-being’, the World Health Organization (WHO) has proposed the “Ending the neglect to attain the Sustainable Development Goals: a road map for neglected tropical diseases 2021–2030” [8] and the “Global Technical Strategy for Malaria 2016-2030” [9]. To attain these objectives, it is essential to implement integrated, multidisease intervention strategies. Collaborative research efforts are crucial to developing cohesive and sustainable public health strategies, which can effectively mitigate disease burdens and facilitate long-term improvements in global health outcomes.

The WHO defines quality of care as the extent to which health services provided to individuals and groups increase the likelihood of achieving desired health outcomes. Health services must be timely, equitable, integrated and efficient to ensure effective, safe and people-centred care. The quality of healthcare has improved, but inequalities remain between regions and countries [10,11]. For instance, there are gender, ethnic, and socio-economic disparities in the disease burden between NTDs and malaria, which may stem from risk factors such as susceptibility due to environmental differences, lack of healthcare knowledge, or inadequate access to convenient screening. Furthermore, although most NTDs predominantly occur in tropical and subtropical regions, global public health issues are often interlinked, and certain NTDs may also affect other regions or spread through travel, migration, and other vectors. Analyzing these diseases from a global perspective helps to comprehensively understand the global health burden of NTDs and malaria, rather than limiting the scope to traditional high-prevalence areas. By incorporating data from around the world, attention can be drawn to the potential burden of NTDs and malaria in regions where they are less common, thereby fostering increased support for public health policies and resource allocation in these areas [12,13].

Theoretically, the higher the burden of disease, the more resources health systems need to allocate [14]. In reality, regions with high disease burdens are mostly economically underdeveloped, and higher costs may reduce access to treatment for NTDs and malaria [15]. Inequalities in the distribution of healthcare facilities and resources can slow down the achievement of the goal of eliminating NTDs and malaria [4]. At the same time, these areas may suffer from more disease complications than economically developed areas, further increasing the burden of disease in these areas [16]. Therefore, there is a need to introduce a new index, the quality of care index (QCI), to quantify the quality of care for NTDs and malaria, to analyse and prioritise inequalities by calculating the QCI to visualise regional and national disparities, and to provide recommendations to policymakers to ensure that quality healthcare services are provided to areas of high disease burden. Areas with a higher disease burden should be allocated high-quality healthcare services [14]. There has been no study assessing the QCI for NTDs and malaria. In this study, we aimed to use the Global Burden of Disease Study 2021 (GBD 2021) to compare inequalities in quality of care for NTDs and malaria by calculating the QCI, globally, regionally, and nationally across gender and different age groups, in 1990 and 2021.

## Materials and methods

### 1. Overview and data sources

In this study, the GBD 2021 from 1990 and 2021 presented in ‘GBD compare’ and Institute for Health Metrics and Evaluation website were retrieved. In the International Classification of Diseases, 10th Revision (ICD-10) system, NTDs and malaria are documented under diagnosis codes, which are presented in S1 Table [17]. This study is in accordance with the Guidelines for Accurate and Transparent Health Estimates Reporting (GATHER) [18].

### 2. Quality of care index

To assess QCI, we created four sub-indicators from the six primary indicators; for diseases with missing mortality and morbidity data, two sub-indicators were constructed from the four primary indicators. These indicators are the mortality to incidence ratio (MIR), the disability-adjusted life year (DALY) to prevalence ratio, the prevalence to incidence ratio, and the ratio of years of life lost (YLL) to years lived with disability (YLD). We selected the overall disease category of NTDs and malaria. NTDs include leishmaniasis, cystic echinococcosis, dengue, rabies, intestinal nematode infections (including ascariasis, trichuriasis and hookworm disease), leprosy, and other NTDs. Principal component analysis (PCA) was performed on the four or two obtained secondary indicators, with the first principal component extracted being defined as the QCI. It was then converted into a value ranging from 0 to 100 according to the following formula. PCA is a statistical technique that combines multiple correlated indicators into a single composite score by capturing the maximum variance in the data, thereby simplifying complex multidimensional information into one representative index [19].


$$QCI=\frac{QCI(x)-{QCI}_{\min}}{{QCI}_{\max}-{QCI}_{\min}}$$

### 3. SDI stratification

The Socio-Demographic Index (SDI) is a composite indicator used in the GBD 2021 to measure the level of socio-demographic development in a country or region. The SDI is based on three key factors: per capita income, average educational attainment, and total fertility rate. The standardized rankings of these three factors are averaged to produce a value between 0 and 1, where 0 indicates the lowest level of socio-demographic development and 1 indicates the highest. Based on the SDI values, countries and regions worldwide are divided into five categories: high SDI, high-middle SDI, middle SDI, low-middle SDI, and low SDI.

### 4. Age and gender differences

The GBD 2021 includes multiple age groups, ranging from 5-year, 10-year, 15-year, to 20-year intervals, among others. Given that age influences incidence rates, mortality, and disease burden, this study selects 5-year age groups to capture these variations with greater precision, thereby reflecting the dynamic changes in disease burden across different age cohorts. We selected age groups in the GBD 2021 with five-year interval as an age group, ranging from birth to 95 + years, resulting in a total of 20 age groups. By calculating the QCI for each age group globally and at different SDI levels, we compared the differences in the quality of care across regions. Additionally, by calculating the QCI at the gender level for each country and region, and determining the gender disparity ratio (GDR), we observed gender inequality. The GDR is calculated using the following formula.


$$GDR=\frac{QCI(F)}{QCI(M)}$$

The GDR greater than 1 indicates that women are more likely to receive better quality care, while the GDR less than 1 indicates that men are more likely to receive better quality care.

### 5. Statistical analysis

Incidence, prevalence, mortality, DALYs, YLLs and YLDs are reported as 95% uncertainty intervals (UI). Estimates of change and trends are significant when the UIs do not overlap over the time period. All statistical analyses, figures, and data in this study were done by R v4.4.0.

## Results

### 1. Neglected tropical diseases and malaria

#### 1.1. Overview.

In 2021, the total number of new cases of NTDs and malaria was 309,802,762, with 847,472 global deaths attributed to these conditions. NTDs and malaria led to 71,629,913 DALYs in 2021, compared to 87,421,645 DALYs globally in 1990. Compared to 1990, the age-standardized incidence rates (ASIR) of NTDs and malaria increased in high SDI, high-middle SDI, and middle SDI regions in 2021, while significantly decreasing in low SDI and low-middle SDI regions. In 2021, the age-standardized mortality rate (ASMR) was 0.05 in high SDI regions and 50.04 in low SDI regions (S2 Table).

#### 1.2. Quality of care index.

**Fig 1:**
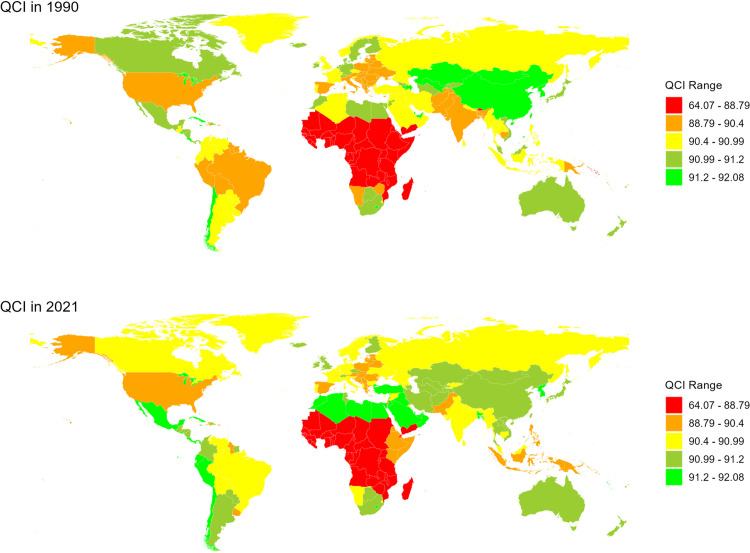
The QCI of the NTDs and malaria in both genders by country in 1990 and 2021. The maps show the global distribution of QCI values in 1990 (top) and 2021 (bottom). Green indicates a higher QCI, while red indicates a lower QCI. The variation in QCI reflects global disparities in healthcare quality and access. Republished from Global country administrative boundary data under a CC BY license, with permission from the Institute of Geographical Sciences and Natural Resources Research, original copyright 2014-2024.

**Fig 2:**
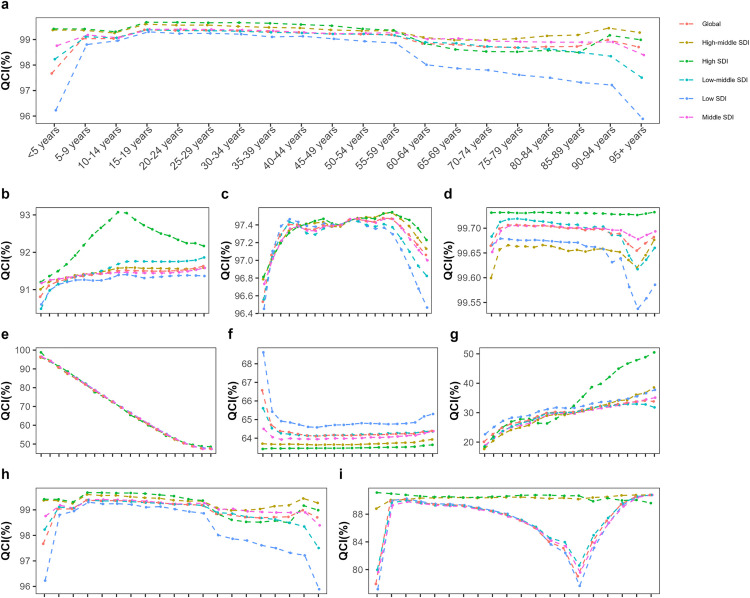
Plot of age trends in QCI NTDs and malaria in 2021. NTDs and malaria **(a)**, leishmaniasis **(b)**, cysticercosis **(c)**, dengue **(d)**, rabies **(e)**, intestinal nematode infections **(f)**, leprosy **(g)**, other NTDs (h) and malaria **(i)**. The QCIs are expressed on a scale of 1 to 100, with each line indicating the relationship between QCI and age in different regions (SDI quintile regions and global regions).

In 2021, the QCI for NTDs and malaria was 88.3. The QCI differences between females and males were not significant, ranging between 88 and 90 (Fig 1). The QCI increased with age, with the fastest growth observed before the age of 9 (rising from 71.9 to 90.0). After the age of 10, the QCI stabilized at around 90. Compared to 1990, the QCI in all SDI regions changed slightly in 2021, with the biggest changes seen in the under-5 age group. For example, in low SDI regions, the QCI increased from 59.7 to 62.6, in low-middle SDI regions, it decreased from 82.4 to 79.2, and in middle SDI regions, it declined from 86.0 to 84.1 (S3 Table). In all SDI regions, the QCI reached 90 in the 5–9 age group and then stayed close to 90 with some small changes. Except in low SDI regions, there was a decline in the QCI for the 70–74 age group (from 88.1 in 1990 to 85.3 in 2021) (Fig 2a).

#### 1.3. Gender disparity ratio.

**Fig 3:**
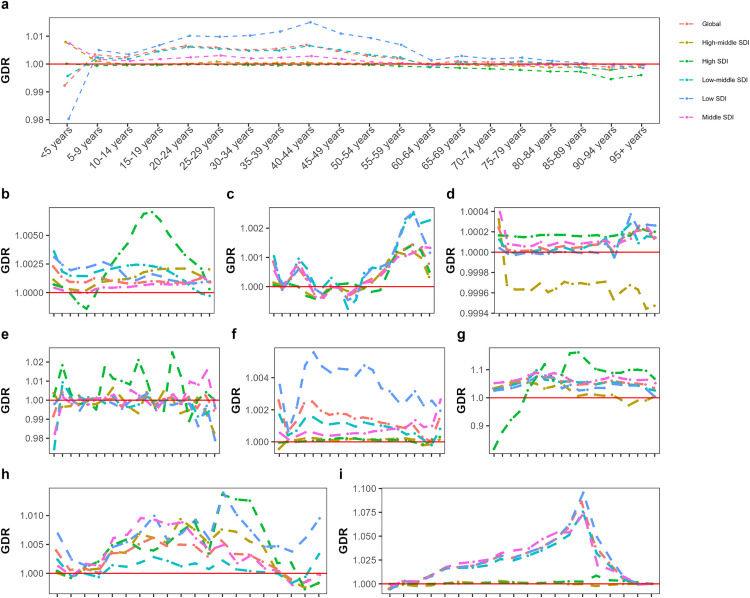
The association between gender disparity ratio and age for all NTDs and malaria globally and by SDI quintile regsions in 2021. This figure demonstrates estimates for both genders combined. Each line shows the association between GDR and age in different areas including SDI quintile regions and global region. Range of GDR is represented in 9 figures for diferent diseases, including NTDs and malaria **(a)**, leishmaniasis **(b)**, cysticercosis **(c)**, dengue **(d)**, rabies **(e)**, intestinal nematode infections **(f)**, leprosy **(g)**, other NTDs (h) and malaria **(i)**.

**Fig 4:**
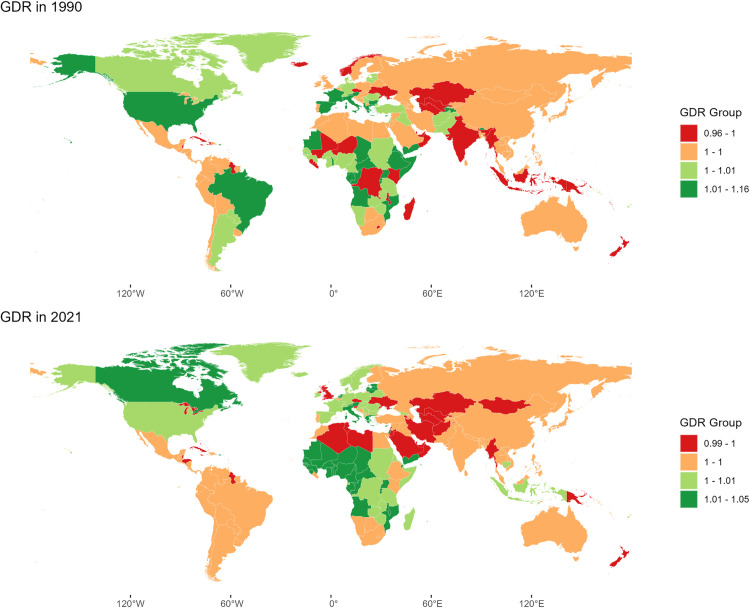
Age-standardized global map of gender disparity ratio in 1990 and 2021. The GDR is QCI score for females divided by QCI score for males, so that higher scores represent better care in females and lower scores represent better care in males. This figure illustrates the GDR in different countries and territories. Republished from Global country administrative boundary data under a CC BY license, with permission from the Institute of Geographical Sciences and Natural Resources Research, original copyright 2014-2024.

In 1990 and 2021, the global GDR values were 1.00 and 1.01, respectively. Globally, the GDR was highest in the 40–44 age group, fell below 1 in those over 80 years old, and was lowest in the 25–29 age group, indicating better quality of care for females in 2021. In both 1990 and 2021, the GDR across SDI regions ranged from 0.99 to 1.01. In 2021, the age-related GDR trends in different SDI regions were mostly similar to the global trend. However, in high-middle and middle SDI regions, the GDR was over 1 for children under 5, while in low SDI regions, it peaked in the 40–44 age group (Fig 3a). In 1990, the lowest GDRs were in Niger (0.96), Sierra Leone (0.99), and Kenya (0.99), while the highest were in the Cook Islands (1.16), Malta (1.08), and Djibouti (1.05). In 2021, the highest GDRs were in Burkina Faso (1.05), Mozambique (1.05), and Monaco (1.04), but the lowest were in Papua New Guinea, Oman, and New Zealand (Fig 4).

### 2. Neglected tropical diseases

#### 2.1. Leishmaniasis.

In 1990, the number of leishmaniasis cases was 1,011,925, increasing to 1,096,860 in 2021. In 2021, leishmaniasis caused 781,188 DALYs, compared to 4,670,788 DALYs globally in 1990 (S2 Table). Compared to 1990, the ASIR of leishmaniasis decreased in all SDI regions in 2021, except in middle SDI regions, where it increased from 15.41 in 1990 to 24.54.

The QCI for leishmaniasis was 91.3. In 2021, the highest QCIs were in El Salvador, Egypt, and Turkmenistan, at 94.0, 93.0, and 92.4, respectively, while the lowest were in Zambia, Angola, and Bhutan, at 56.8, 62.8, and 63.5, respectively. Between 1990 and 2021, QCI increased in 86 out of 107 countries and regions, with the greatest improvements in South Sudan, Djibouti, and Ethiopia, increasing by 36.9, 13.6, and 13.3, respectively (S3 Table). The GDR for leishmaniasis is shown in the chart. At the national level, except for Bangladesh (0.93) and Bhutan (0.96), the GDR is around 1.0, indicating nearly equal care conditions for both genders globally (Fig 3b).

#### 2.2. Cystic echinococcosis.

In 1990, the number of cystic echinococcosis cases was 90,312, increasing to 148,521 in 2021. In 2021, cystic echinococcosis caused 105,072 DALYs, compared to 258,248 DALYs globally in 1990 (S2 Table). Compared to 1990, the ASIR of cystic echinococcosis increased in high SDI and middle SDI regions in 2021, while decreasing in the other three SDI regions. In 2021, the ASMR in low SDI regions was 0.24. The QCI for cystic echinococcosis was 97.4. Between 1990 and 2021, the QCI increased in all 210 countries and regions, with only slight improvements, remaining around 97, indicating a high quality of care for cystic echinococcosis (S3 Table).

#### 2.3. Dengue.

In 1990, the number of dengue cases was 26,447,129, which increased to 58,964,185 in 2021. In 2021, dengue caused 2,076,525 DALYs, compared to 1,248,669 DALYs globally in 1990 (S2 Table). Compared to 1990, the ASIR of dengue increased in all SDI regions in 2021, except for the middle SDI region, where it rose from 782.97 in 1990–1,269.27. In 2021, the ASMR increased in all regions except high SDI regions, with the highest rate in low-middle SDI regions at 1.18. The overall age-standardized QCI for dengue was 99.7. From 1990 to 2021, 65 out of 132 countries and regions saw an increase in QCIs, though the changes were relatively small. The Cook Islands had the biggest improvement, with a 20.5 point rise since 1990 (S3 Table). The QCI for dengue varied by age group but remained above 99 (Fig 2d).

#### 2.4. Rabies.

In 1990, the number of rabies cases was 22,035, which decreased to 10,181 in 2021. In 2021, rabies caused 569,550 DALYs, compared to 1,368,780 DALYs globally in 1990 (S2 Table). The ASIR of rabies decreased in all regions except for high SDI regions, where it increased. The pattern of ASMR was similar. The QCI for rabies was 77.8. Between 1990 and 2021, QCI decreased in 179 out of 210 countries and regions, with the greatest declines in Lithuania, Latvia, and Peru, which fell by 36.2, 31.2, and 29.1, respectively. The largest increases were in Canada and Germany, with increases of 23.7 and 20.3, respectively (S3 Table). Across all age groups, the QCI for rabies shows a trend of gradually decreasing with age, with the highest QCI in the under-5 age group and the lowest in the 95 + age group (Fig 2e).

The quality of care for rabies across 210 countries and regions reveals sex differences. Germany, the Bahamas, and Canada had the highest GDRs, at 1.58, 1.55, and 1.50, respectively, indicating that females receive better care than males in these areas. Conversely, the Maldives, Iraq, and Austria had the lowest GDRs, at 0.66, 0.72, and 0.73, respectively, showing that males receive better care than females in these regions (S3 Table). Compared to 1990, the GDR for countries and regions still shows significant gender disparities (Fig 3e).

#### 2.5. Intestinal nematode infections.

In 1990, the number of intestinal nematode infections was 1,563,530,463, which decreased to 642,720,070 in 2021. In 2021, intestinal nematode infections caused 1,381,641 DALYs, compared to 5,994,628 DALYs globally in 1990 (S2 Table). Compared to 1990, the incidence rates decreased in all SDI regions. The QCI for intestinal nematode infections was 64.5. Between 1990 and 2021, QCI decreased in 138 out of 152 countries and regions, with the greatest declines in Oman, the United Arab Emirates, and Equatorial Guinea, decreasing by 8.3, 6.8, and 5.6, respectively (S3 Table). Across all age groups, the QCI for intestinal nematode infections shows a trend of gradually decreasing with age (Fig 2f).

#### 2.6. Leprosy.

In 1990, the number of leprosy cases was 543,622, which decreased to 408,776 cases in 2021. In 2021, leprosy caused 21,428 DALYs, compared to 26,775 DALYs globally in 1990 (S2 Table). The QCI for leprosy was 29.7. Between 1990 and 2021, QCI increased in 83 out of 144 countries and regions, with the greatest increases in Mozambique, Madagascar, and Nepal, rising by 8.7, 7.2, and 6.0 points, respectively. Conversely, the largest decreases were observed in Qatar, Tunisia, Comoros, and Saudi Arabia, with reductions of 10.3, 9.0, 8.3, and 8.1 points, respectively (S3 Table). Across all age groups, the QCI for leprosy shows a trend of increasing with age (Fig 2g).

The GDR for leprosy in 144 countries and regions reveals gender differences in care quality. The lowest GDRs were in Oman, Lebanon, and the United Arab Emirates, at 0.40, 0.46, and 0.46, respectively, indicating that males receive better care in these regions. In contrast, higher GDRs were found in New Zealand, Afghanistan, and Kazakhstan, at 2.96, 1.51, and 1.43, respectively, suggesting that females receive better care in these areas (S3 Table). However, within SDI regions, the care quality for leprosy is relatively equal between genders, with GDRs generally around 1, indicating gender equality in care (Fig 3g).

#### 2.7. Other neglected tropical diseases.

In 1990, the number of cases of other NTDs was 84,530,285, which increased to 103,762,442 in 2021. In 2021, these diseases caused 4,218,302 DALYs, compared to 3,528,752 DALYs globally in 1990 (S2 Table). Compared to 1990, the ASMR increased in high SDI and low-middle SDI regions, while decreasing in the other regions. The QCI for other NTDs was 98.8. Between 1990 and 2021, QCI increased in 149 out of 210 countries and regions, with the greatest increases in Guatemala, São Tomé and Príncipe, and Guinea, rising by 6.2, 5.5, and 3.4, respectively (S3 Table).

### 3. Malaria

In 1990, the global number of new malaria cases was 217,409, which increased to 249,117 in 2021. In 2021, malaria caused 55,174,061 DALYs, compared to 57,888,053 DALYs globally in 1990 (S2 Table). Compared to 1990, the ASIR of malaria decreased across all SDI regions in 2021. In 2021, the ASMR for malaria was 0.01 in high SDI regions and 46.05 in low SDI regions.

The QCI for malaria was 87.0. In 2021, the highest QCIs were in Thailand, Panama, and Vietnam, at 90.9, 90.8, and 90.8, respectively, while the lowest were in Rwanda, the Dominican Republic, and Haiti, at 80.1, 78.5, and 78.3, respectively. Between 1990 and 2021, 66 out of 88 countries and regions saw an increase in QCI, with Haiti, Senegal, and Djibouti showing the greatest improvements, increasing by 10.9, 7.3, and 6.7. The QCIs varied from 90.6 in high SDI regions to 86.7 in low SDI regions, with the general trend showing that QCI improves as economic levels rise (S3 Table). The QCI peaked in the 95 + age group (90.8) and was lowest in the under-5 age group (78.0) (Fig 2i).

## Discussion

Among the QCI estimates for different causes, this study provides quality of care for NTDs and malaria. Previous studies have provided QCI for haematological malignancies [20], colorectal cancer [21], thyroid cancer [22], endocarditis [23], cirrhosis [24], and bipolar disorder [25]. We systematically assessed the global burden of disease and quality of care for NTDs and malaria by calculating QCI. Our data were obtained from the GBD 2021 data, which provided evidence to guide improvements in specific areas. Findings highlight differences in care over time by gender, age, and geographic region.

Analysis of key epidemiological indicators for NTDs and malaria shows that the number of new cases of malaria, leishmaniasis, cysticercosis, dengue and other NTDs has increased in 2021 compared to 1990, possibly related to the climate change [13], population migration and urbanization, and increasing drug resistance [26,27]. Except for dengue, which showed an increase in DALYs, the burden of most other NTDs and malaria declined during the study period. The rise in dengue burden may be partly attributed to global climate change, which creates more favorable conditions for *Aedes aegypti*—the primary vector of dengue—to survive and reproduce [28]. In contrast, the decrease in malaria burden likely reflects intensified control efforts, including expanded access to effective prevention measures and treatments, improved surveillance, and strengthened health policies. Furthermore, poor sanitation and limited access to clean water [29,30] create environments conducive to *Aedes* mosquito breeding, thereby facilitating dengue transmission [31]. The results suggest a positive association between disease burden and economic status. Consistent with our findings, a study by Dirk and colleagues [1] found that NTDs, which have been neglected on the global health agenda, are strongly associated with poverty and contribute to a heavy local disease burden.

Research on QCI for diseases is crucial, especially in the areas of global health, public health and health policy, and the QCI provides a standardised way of measuring and comparing the quality of healthcare in different regions and countries, identifying health disparities between regions, age groups and genders, and is an important indicator for assessing the effectiveness of health policies and interventions [20]. The quality of care for NTDs and malaria is closely linked to global health goals, such as the United Nations SDG of ‘health well-being’ [32].

Clinical trials have shown that timely and standardised drug treatment can significantly reduce mortality and complications of NTDs and malaria [33]. For example, the use of anti-malarial drugs, such as artemisinin combination therapies, has dramatically reduced the number of severe illnesses and deaths from malaria [34]. For NTDs such as leishmaniasis [35] and dengue [36], studies have shown that improved treatment can be effective in reducing the long-term disability and burden of disease [37]. In addition, achieving early diagnosis and intervention for diseases can improve prognosis and reduce the burden of disease [38]. The use of rapid diagnostic tests has increased the early detection of malaria, enabling timely treatment and reducing the overall disease burden. Additionally, it helps avoid unnecessary treatment in malaria-negative cases, thereby improving treatment efficiency. Similarly, increased early detection and treatment of tropical diseases such as rabies, and intestinal nematode infections can reduce disease transmission and burden [39]. Additionally, mass drug administration (MDA) programs for NTDs, like intestinal nematode infections, trachoma and schistosomiasis, have successfully reduced the prevalence and intensity of these diseases in many high-endemic areas [40,41], demonstrating the effectiveness of improving care quality and preventive measures. In several African countries, improved community health worker programs have significantly increased the timely treatment rates for malaria patients and reduced child mortality [42]; These efforts, along with other malaria control strategies such as insecticide-treated bed nets and indoor residual spraying, have played a crucial role in reducing the burden of the disease over the past few decades [43]. Economics studies have shown that interventions to improve the quality of care not only reduce the burden of disease but are also cost-effective [44]. For example, the provision of a wide range of malaria prevention and treatment interventions reduces economic losses due to disease, including reduced productivity and increased health-care costs [45]. Such cost-effectiveness analyses provide strong evidence support for policymakers. High-quality healthcare facilitates the early detection and timely treatment of diseases, thereby reducing the further spread and progression of illness. In addition, improvements in care quality enhance patient adherence to treatment regimens and overall health management. Through the provision of targeted patient education and support, healthcare providers can improve patients’ understanding and compliance with preventive and therapeutic measures, thereby effectively lowering the disease burden at the population level. Furthermore, the enhancement of care quality also involves the strengthening of preventive interventions, such as promoting health education and controlling environmental risk factors (e.g., vector control), which are essential in alleviating the burden of NTDs and malaria. All of these evidences suggest that the quality of care is strongly associated with the burden of disease and that improving the quality of care is effective in reducing the burden of disease. Quantifying the quality of care by studying the QCI can provide a strong basis for improving the quality of care and formulating relevant policies.

By analysing the results of the study, we found that QCI was higher in high SDI areas, which suggests that economic level may influence the quality of care for diseases. In addition, we conducted a detailed analysis of the countries with higher QCIs and found that Honduras, Viet Nam, Panama, and Thailand had relatively high QCI values in the case of malaria. Upon further investigation, we found that these countries have received strong support for malaria control from both their governments and international organizations, and have implemented large-scale malaria control and elimination programmes [46], which have helped improve the quality of care. Secondly, these countries have invested in public health infrastructure over the past decades, and have established relatively well-developed primary healthcare systems that are able to detect, diagnose, and treat malaria in a timely manner and ensure that patients receive high-quality care [47]. For example, Vietnam’s network of community health workers has played a key role in malaria control and ensures that people living in remote areas are treated in a timely manner [48]. In addition, these countries have established comprehensive malaria surveillance and information systems, which enable them to track malaria epidemiological trends in a timely manner and respond rapidly to emergencies, making malaria prevention and treatment more effective and thus improving the quality of care.

On the other hand, NTDs and malaria mainly affect tropical and subtropical regions of the globe, especially in low- and middle-income countries [39]. In our study, countries such as Zambia, Angola, Bhutan, and Bangladesh had low QCI (in the case of leishmaniasis), a phenomenon that may be attributed to inadequate basic sanitation, lack of capacity for early diagnosis and detection, insufficient public health interventions, economic deprivation and unequal distribution of health resources, and the environment [49,50]. When comparing the raw data downloaded from GBD 2021 with the data from the World Health Organization (WHO), we observed discrepancies in the disease burden estimates. This could be attributed to differences in the methodologies used by GBD and WHO, as well as the timing of the interventions and data collection periods. For example, the WHO may have more up-to-date information from ongoing health programs or more region-specific data that is not yet incorporated into the GBD database. Despite these differences, the GBD study remains a valuable tool for understanding global health trends and provides credible estimates that inform policy decisions, as it offers a broad, global perspective on disease burden and healthcare quality. Simultaneously, this indirectly corroborates the feasibility and accuracy of the approach that involves large-scale investments in health infrastructure in low- and middle-income countries, increased research and development of vaccines and treatments for NTDs and malaria, particularly to address drug resistance, the establishment and refinement of disease surveillance systems, enhanced international cooperation and resource sharing [51], improved public health education, heightened awareness of NTDs and malaria [14], and the equitable distribution of treatment resources [47]to reduce regional disparities in QCI.

Examining the trend of QCI in different age groups, we found that the QCI for rabies and intestinal nematode infections both showed a decreasing trend with age. For rabies, this trend may be attributed to the lower risk of infection in older age groups, as they are less likely to be exposed to infected animals or to engage in behaviors that increase the risk of animal bites. Additionally, older individuals may have increased awareness of personal safety, further reducing their exposure to potential sources of infection. In contrast, the decreasing trend in QCI for intestinal nematode infections could be related to improvements in hygiene and sanitation as individuals age. Older individuals may have better access to healthcare and may practice more consistent hygiene behaviors, such as frequent handwashing and avoiding consumption of contaminated food, which reduces their exposure to intestinal parasites [52,53]. Second, public health policies and interventions often prioritize child populations. For instance, in the case of rabies, vaccination and safety education are commonly intensified for children [54], while school health projects and nutritional interventions are typically directed at addressing intestinal nematode infections in this group. Additionally, children benefit from a greater degree of protection from the less vigilant health behaviors of adults. Health services may also be more focused on children, particularly in remote or resource-limited areas, where health resources are often disproportionately allocated to child-focused programs [55]. Moreover, the symptoms of these diseases may not be as pronounced or immediately affect quality of life in adults as they do in children, leading to a tendency among adults to delay early treatment or preventive measures. This delayed response may contribute to a lower QCI in the adult population. Beyond these two diseases, the variation in QCI by age shows a pattern of lower scores in children under five years and a subsequent decline after the age of 65. The first contributing factor is the incomplete development of the immune system in young children and the decline in immunity in older adults [56]. Additionally, older adults often face limited access to healthcare resources, especially in aging populations and areas with significant resource constraints. The healthcare system may become overburdened [57,58], further lowering QCI, compounded by the complex care needs of the elderly, who often suffer from multiple chronic conditions, which can impair the quality of care for specific infectious diseases [59]. Lastly, inadequate health education, insufficient social support, and a lack of resources for disease prevention and management may contribute to lower QCI in certain populations.

Among the NTDs, we find that gender inequality is more pronounced in leishmaniasis, leprosy and rabies. The manifestation of such gender inequalities in these diseases is linked to a complex set of social, cultural, economic and political factors [60]. Addressing these inequalities requires not only improving women’s access to healthcare, but also eliminating entrenched gender discrimination and social injustice through education, social reform and policy adjustments to achieve true equality in the quality of care.

The main strengths of our study are as follows: first, it is the first study to address the quality of care for NTDs and malaria globally, providing strong indicators for understanding and improving the quality of care for NTDs and malaria. Secondly, our study analyses the causes of differences in quality of care by age, gender and region, providing evidence support for eliminating gender inequalities and regional disparities. However, our study has some limitations. One key limitation is the reliance on the GBD database, which uses national data registries for modelling purposes to address data scarcity. While the GBD database provides valuable estimates, it may not fully capture regional variations or specific local contexts due to incomplete or inconsistent data reporting in some countries. As a result, our findings could be influenced by data gaps, particularly in areas with limited or unreliable national health data. This limitation may impact the accuracy of our results, especially in regions with significant epidemiological or socio-economic differences that are not fully represented in the available data. Second, our study did not include an assessment of ethnic and racial differences, which could provide important insights into disparities in healthcare quality. Ethnic and racial factors can influence health outcomes due to differences in genetic susceptibility, access to care, cultural practices, and socio-economic conditions. Therefore, the absence of this analysis may limit the generalizability of our findings across diverse populations. Third, the accuracy of the QCI could be influenced for diseases with high prevalence but low mortality. For such diseases, the focus is often on managing the chronic or long-term nature of the condition rather than preventing mortality, which may result in variations in the quality of care received by patients. The QCI might not fully capture the complexities of care required for these conditions, potentially leading to an underestimation of healthcare quality in high-prevalence, low-mortality diseases. Fourth, it is worth emphasizing that the QCI mainly captures health outcomes and does not directly assess aspects of healthcare delivery, such as the availability of trained personnel, access to medications, or health facility infrastructure. Additionally, the index may be affected by factors beyond the healthcare system itself, including overall population health, ecological characteristics of diseases, and the accuracy of disease surveillance. Due to the lack of comprehensive data spanning multiple decades and diverse geographic regions, empirical validation of the QCI against direct indicators of healthcare quality remains limited at present. We therefore suggest that future studies focus on validating and refining the QCI as more detailed and reliable data on healthcare delivery become accessible, thereby improving its effectiveness as a measure of healthcare quality.

## Conclusion

This study highlights global disparities in the quality of care for NTDs and malaria, emphasizing the need for targeted resource allocation and the reduction of economic disparities to improve healthcare outcomes. The findings offer a solid foundation for policymakers to develop strategies focused on underserved regions, increasing healthcare funding, and enhancing training for healthcare workers. The QCI presents a valuable tool for monitoring and improving healthcare quality, and its more effective application could guide future interventions. By addressing these disparities, decision-makers can help reduce the burden of NTDs and malaria, ensuring that resources are directed where they are most needed.

## Supporting information

S1 Table ICD, 10th Revision system, codes mapped for Neglected tropical diseases and malaria. (XLSX)

S2 Table The all-ages number and age-standardized rates of incidence, death, disability-adjusted life years(DALYs), years of life lost (YLLs), years lived with disability (YLDs), and prevalence in both sexes globally in 1990 and 2021. Data in parentheses are 95% uncertainty intervals(95% UI).
(XLSX)

S3 Table Sheet 1-Age-standardized QCI Values (%) and GDR for the NTDs in Selected Locations in 1990 and 2021. Sheet 2-Age-standardized QCI Values (%) and GDR for Leishmaniasis in Selected Locations in 1990 and 2021. Sheer3-Age-standardized QCI Values (%) and GDR for Cystic echinococcosis in Selected Locations in 1990 and 2021. Sheet 4-Age-standardized QCI Values (%) and GDR for dengue in Selected Locations in 1990 and 2021. Sheet 5-Age-standardized QCI Values (%) and GDR for Rabies in Selected Locations in 1990 and 2021. Sheet 6-Age-standardized QCI Values (%) and GDR for Intestinal nematode infections in Selected Locations in 1990 and 2021. Sheet 7-Age-standardized QCI Values (%) and GDR for Leprosy in Selected Locations in 1990 and 2021. Sheet 8-Age-standardized QCI Values (%) and GDR for other NTDs in Selected Locations in 1990 and 2021. Sheet 9-Age-standardized QCI Values (%) and GDR for Malaria in Selected Locations in 1990 and 2021.
(XLSX)

## Abbreviations:

NTDs: Neglected Tropical Diseases
QCI: quality of care index
GBD 2021: Global Burden of Disease Study 2021
GDR: gender disparity ratio
DALY: Disability-Adjusted Life Year
YLL: Years of Life Lost
YLD: Years Lived with Disability
ASMR: age-standardized mortality rate
SDI: Socio-Demographic Index
SDG: Sustainable Development Goal
WHO: World Health Organisation
IHME: Institute for Health Metrics and Evaluation
ICD-10: International Classification of Diseases, 10th Revision
GATHER: Guidelines for Accurate and Transparent Health Estimates Reporting
MIR: mortality to incidence ratio
PCA: Principal component analysis
UI: uncertainty intervals
ASIR: age-standardized incidence rates
MDA: mass drug administration.
